# Earwig Crawling in the Ear: Myth or Truth

**DOI:** 10.7759/cureus.14827

**Published:** 2021-05-03

**Authors:** Hamin Jeong, Jung Eun Shin, Chang-Hee Kim

**Affiliations:** 1 Otorhinolaryngology, Konkuk University Medical Center, Seoul, KOR

**Keywords:** dermaptera, earwig, otalgia, tinnitus

## Abstract

A 24-year-old man presented to our outpatient clinic with the left tinnitus and otalgia, which had awakened him early in the morning. Otoendoscopic examination revealed an earwig crawling in the external auditory canal. The earwig was carefully taken out with ear forceps. The tympanic membrane and external auditory canal were normal without traumatic lesions, and audiometric testing revealed normal hearing. Earwigs are insects of the order Dermaptera, and the name "earwig" originated from an ancient superstition that earwigs burrow through the external auditory canal and eat sleeping persons’ brains. Although this superstition turned out to be unfounded, the earwigs sometimes do enter the ear.

## Introduction

There has been an old superstition that earwigs may crawl into the human ears and lay their eggs. The Merriam-Webster’s Dictionary defines an earwig as “any of numerous insects (order Dermaptera) having slender many-jointed antennae and a pair of cerci resembling forceps at the end of the body” [1]. The Encyclopedia Britannica describes that the name “earwig” is derived from the Anglo-Saxon word meaning “ear creature,” probably because of a widespread ancient superstition that earwigs crawl into the ears of sleeping people [2]. Although the above-mentioned superstition about earwigs has been found to be groundless in the modern world, the present study reports a case of the crawling earwig in the ear of a 24-year-old man.

## Case presentation

**Figure 1:**
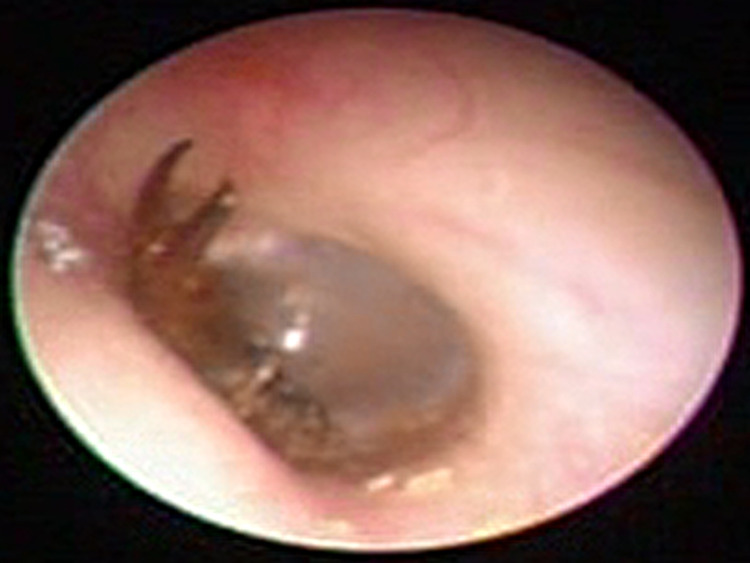
An earwig is crawling in the external auditory canal

A 24-year-old man presented to the outpatient clinic with the left tinnitus and severe otalgia, which had awakened him early in the morning. He was very agitated and reported that a bug seemed to enter into his left ear. Otoendoscopic examination revealed an earwig crawling in the external auditory canal (Figure 1, Supplemental video 1). A brilliant illumination was flashed to attract the insect out of the ear, but it kept crawling in his external auditory canal. So, the earwig was carefully taken out with ear forceps. After taking out the earwig, the ear symptoms were relieved. An otoendoscopic examination showed the normal tympanic membrane and external auditory canal. A pure tone audiometry and tympanometry revealed no abnormality.

## Discussion

The earwig is a nocturnal creature and generally herbivorous [2,3]. The earwigs prefer living in moist and musty places, and are known to occasionally creep into homes being attracted to light. The size varies from 5 to 50 mm in length, and an outer covering is shiny and dark-colored. It has a pair of horny plier-like tail filaments at the posterior end of the abdomen, which is assumed to serve some purpose in mating and defensive action.

There is no consensus on the etymology of the earwig. Due to a long-standing European superstition that earwigs burrow into human brains through the ear canals of sleeping people, the name, earwig, is derived from the Old English ēare, which means "ear," and wicga, which means "insect," or, literally, “beetle” [4]. Although this old superstition is no longer believed to be a truth in the modern world, there have been anecdotal reports that earwigs may climb into the ears [5,6]. However, there has been no report demonstrating an earwig crawling within the ear canal.

Considering that clinicians meet many patients who have insects in their ears at the clinic, the present case may not be that of a rare case. However, the present study aimed to highlight the old superstition about earwigs, which is, to the best of our knowledge, the first study that shows video demonstration of an earwig crawling in the human ear. 

## Conclusions

Although an ancient myth that earwigs burrow through the external auditory canal and eat sleeping persons’ brains is considered unfounded, these bugs sometimes do enter the ear, causing severe ear discomfort.
